# Association Between Dominant-Shoulder Internal-Rotation Restriction and Low-Back Pain in Elite Male High School Soft Tennis Players

**DOI:** 10.7759/cureus.68081

**Published:** 2024-08-29

**Authors:** Toru Tanabe, Hiroichi Miaki, Takumi Watabu, Tomonari Sugano, Katsunori Mizuno, Hitoshi Asai

**Affiliations:** 1 Department of Physical Therapy Rehabilitation, Fukui General Clinic, Fukui, JPN; 2 Graduate School of Pharmaceutical and Health Science Comprehensive Research Course, Kanazawa University, Kanazawa, JPN; 3 Institute of Medical, Pharmaceutical and Health Sciences, Kanazawa University, Kanazawa, JPN; 4 Department of Rehabilitation, Faculty of Health Science, Fukui Health Science University, Fukui, JPN; 5 Department of Orthopedics, Fukui General Hospital, Fukui, JPN

**Keywords:** elite teams, stroke movements, internal rotation restriction, hip, shoulder, multiple logistic regression, male, high school, soft tennis

## Abstract

Background: The incidence of low-back pain (LBP)is high among elite male high school soft tennis players. A previous report showed that hip internal-rotation (IR) restriction in the non-dominant leg could increase the risk of LBP. Moreover, IR in the dominant shoulder is important for serve and stroke movements, suggesting that IR restriction in the dominant shoulder can contribute to LBP. The simultaneous incidence of non-dominant-hip IR restriction may help in developing a good LBP-related factor model; therefore, this study aimed to investigate the association between dominant-shoulder IR restriction and LBP in elite male high school soft tennis players.

Design: This is a single-center cross-sectional study.

Methods: This study included 176 male high school soft tennis players from 14 elite teams. The main measures were IR and external-rotation range of motion (ROM) in the dominant and non-dominant shoulders and hip, assessed using a goniometer with a bubble attached. Multiple logistic regression analyses were performed with the presence of LBP as a categorical variable (LBP, 1; non-LBP (NLBP), 0). Multiple logistic regression models were created as follows: Model 1 included dominant-shoulder IR restriction (loss of glenohumeral IR (GIRLoss)), Model 2 included non-dominant-hip IR ROM, and Model 3 included both GIRLoss and non-dominant-hip IR ROM as the main explanatory variables to test the association between dominant-shoulder IR restriction and LBP, compare its suitability as an associated factor with non-dominant-hip IR restriction, and determine if either or both should be considered in an LBP association model based on model fit. The significance of each multiple logistic regression model was examined using the model χ^2^ test, and a model with P<0.05 was judged as a significant model. Model fit was examined using the Hosmer-Lemeshow test, and P≥0.05 was judged as a good model fit. The Akaike Information Criterion (AIC) and Bayesian Information Criterion (BIC) were used to compare the goodness-of-fit among multiple logistic regression models.

Results: The LBP and non-LBP groups comprised 59 and 117 players, respectively. GIRLoss in the dominant shoulder was a significant independent factor (odds ratio: 1.06, 95% CI: 1.02-1.09; P<0.01) in Model 3, which included hip IR restriction in the non-dominant leg (odds ratio: 0.90, 95% CI: 0.86-0.94; P<0.01). In all models, the explanatory variables fitted were significantly associated with LBP, indicating that the models were significant and fitted well: the AICs (and BICs) for Models 1, 2, and 3 were 198.4 (207.6), 178.6 (187.9), and 168.8 (181.1), respectively, indicating that Model 3 was the best fit.

Conclusions: In elite male high school soft tennis players, IR restriction in the dominant shoulder was associated with LBP. IR restriction in the dominant shoulder and non-dominant hip as a significant factor for LBP may contribute to developing an optimal multiple logistic regression model for LBP.

## Introduction

Asia has numerous soft tennis players [1]. Soft tennis was featured as an official event in the 2023 Asian Games (hosted by the Asian Olympic Council), with participation from 45 countries [2]. It has emerged as an extremely popular sport among Japanese high school students [1], with some high school elite athletes advancing to competition levels and continuing their careers as professional players [3].

Sports activity-related low-back pain (LBP) occurs in 24-36% of adolescents, including high school students [4], reducing athletic performance and limiting participation in training and competitions [5], negatively impacting an athlete's progression to a professional athletic career in the long term [5]. The incidence of LBP among high school soft tennis players is 28% and 14% among male and female players, respectively [6]. Considering the detrimental effect of LBP on an athlete's future sports career, especially among male players, appropriate conditioning and LBP prevention-based strategies should be established for elite male high school soft tennis athletes. A previous report showed that hip internal-rotation (IR) restriction in the non-dominant leg (the leg opposite the upper hand that grasps the racket: steps forward in a forehand stroke) was significantly associated with LBP in elite male high school soft tennis players. Specifically, for every 1° decrease in IR range of motion (ROM) in the non-dominant hip, the risk of LBP increased 1.1-fold [3]. IR motion in the non-dominant hip plays a role in efficiently transferring energy from the lower limb to the upper limb during hitting and throwing movements [7-10]. Therefore, IR restriction in the non-dominant hip may cause LBP due to compensatory loading on the lower back during stroke and serve movements [3].

However, previous studies have not verified the association between LBP and IR restriction in the dominant shoulder, which occurs frequently in lawn tennis and baseball pitchers [11,12]. In lawn tennis and baseball pitching, the characteristic frequent occurrence of IR restriction in the dominant shoulder is associated with contracture of the posterior rotator cuff muscles and posterior shoulder joint capsule and with adaptive changes in the bone due to an increased humeral posterior torsional angle [11-13]. In lawn tennis, the angular velocity of the IR in the dominant shoulder becomes the greatest throughout the phase just before the impact of the serve or forehand stroke [9,14]. Furthermore, the IR of the dominant shoulder plays a crucial role in managing the rotational forces transferred through the kinetic chain during stroke movements, helping to distribute the load more evenly across the body, including the lower lumbar spine [15]. If there is a restriction in the dominant shoulder's IR, the body may compensate by increasing the rotational load on other structures, such as the lower back, to achieve the necessary ROM during these movements. This compensatory mechanism can lead to increased stress on the lower back tissues and ultimately contribute to the development of LBP. Regarding the throwing motion, players with IR restriction in the dominant shoulder have been observed to exhibit a larger trunk rotation angle on the non-dominant side during the ball release phase [16]. This suggests that when the IR motion in the dominant shoulder is limited, the body compensates by excessively rotating the trunk, placing additional stress on the lower back.

The serve and stroke kinematics of soft tennis are similar, where the serve and stroke kinematics closely resemble those in lawn tennis [17] and IR restriction in the dominant shoulder likely increases the rotational load on the lower back, potentially leading to LBP. Therefore, it was hypothesized that IR restriction in the dominant shoulder is associated with LBP in elite male high school soft tennis players. To date, no studies have reported on such a relationship. Therefore, our clinically significant and novel findings may help in establishing a highly compatible LBP association factor model. During stroke and serve movements in tennis, the IR motion of the dominant shoulder reaches its peak just before the ball impact [14]. Considering that IR of the non-dominant hip is a primary motion in transferring energy from the lower to the upper body during these movements, the mechanical contribution of IR motion of the dominant shoulder in the kinetic chain may be inferior to that of the non-dominant hip. While IR of the dominant shoulder is an important factor associated with LBP, its suitability as an associated factor may not extend to IR restriction in the non-dominant hip. However, considering the importance of dominant-shoulder and non-dominant-hip IR restrictions in the kinetic chain, both could be considered relevant factors for LBP, possibly leading to the development of a highly compatible model.

We aimed to clarify the association between dominant-shoulder IR restriction and LBP and validate a multiple logistic regression model with a good fit for LBP, including restriction in IR in the non-dominant hip.

## Materials and methods

Study design

In this observational cross-sectional study, the findings of the survey are described according to the Strengthening the Reporting of Observational Studies in Epidemiology statement [18]. This study adhered to the principles of the Oviedo Convention and Declaration of Helsinki and was approved by the Ethics Review Committee of the Nittaduka Medical Welfare Center (approval number: 2019-56). Informed consent was obtained from parents and coaches because the participants were underage.

Participants

Shoulder and hip rotation ROM were measured by two physical therapists with identical characteristics and at least five years of experience who were certified as athletic trainers by the Japan Sports Association. An assistant (B) described the measurement results. To prevent measurement bias, the measurers and assistant (B) were blinded to the self-administered questionnaire [3]. The order of measurement was random; both dominant and non-dominant shoulders and hips were measured; and all measurements were repeated three times. Specific measurement procedures described in previous studies were followed [3,12,19].

**Figure 1:**
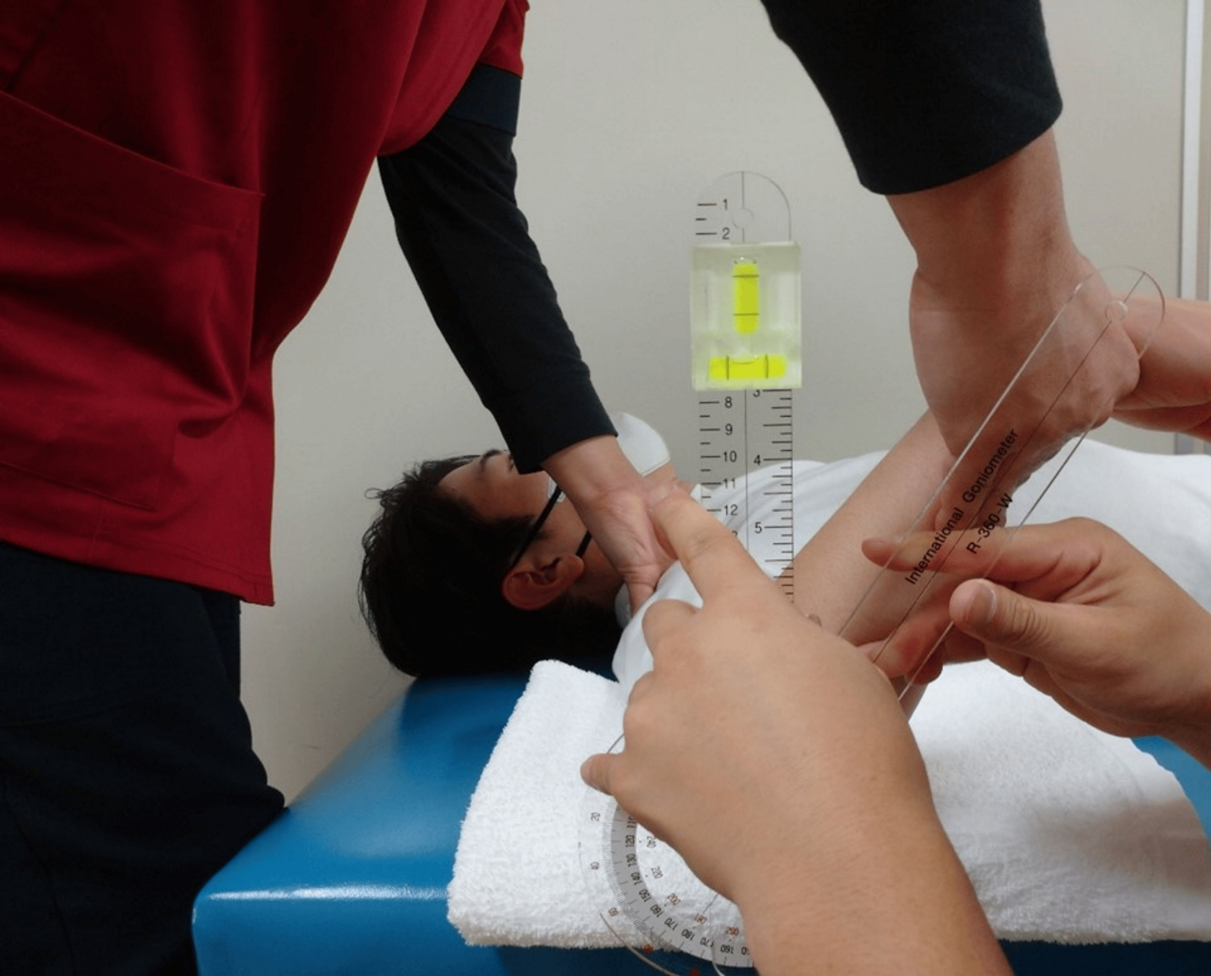
Range of motion measurement of shoulder internal rotation

**Figure 2:**
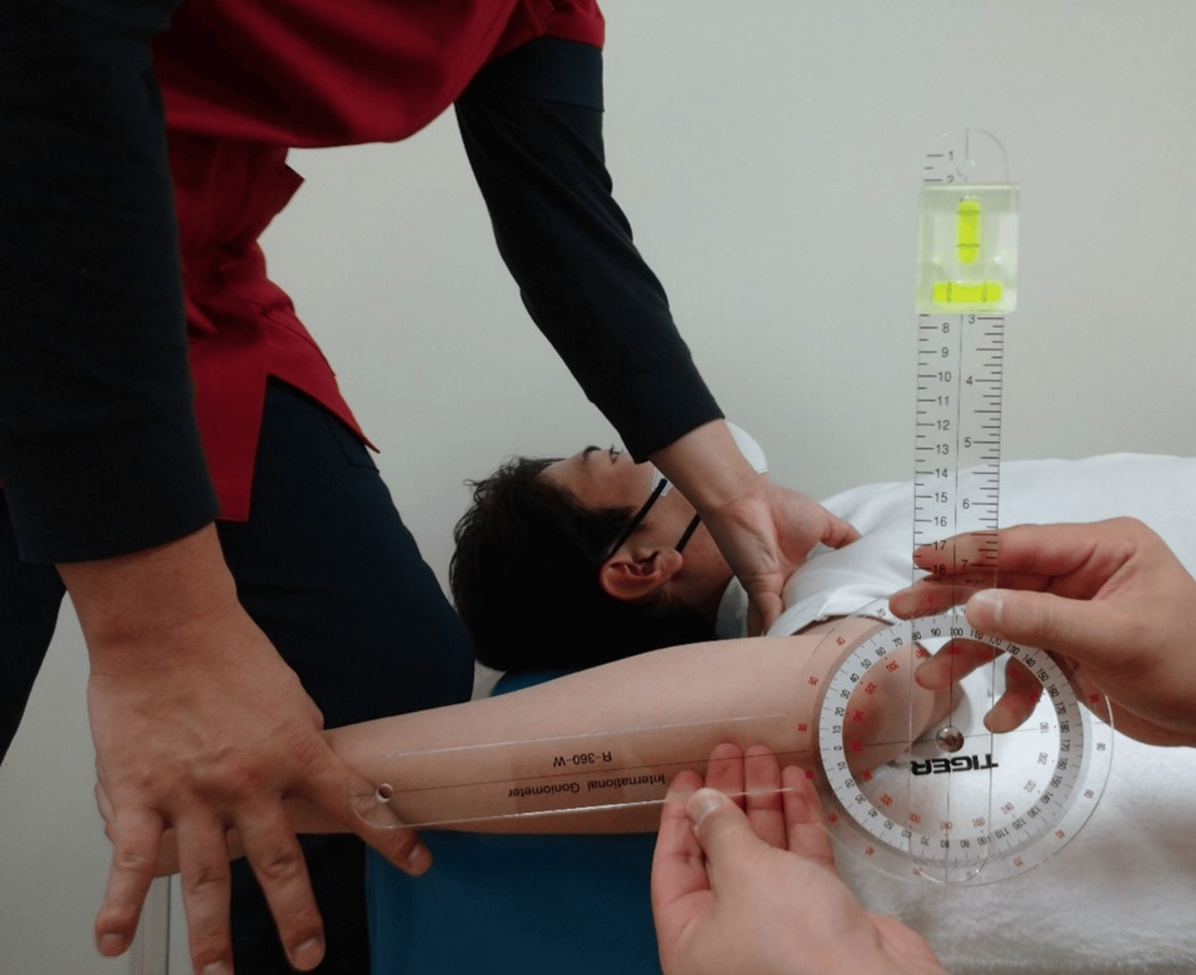
Range of motion measurement of shoulder external rotation

In accordance with prior research, all participants performed a five-minute jogging warm-up followed by self-administered stretching. Stretching was performed on both the dominant and non-dominant sides, with a particular focus on shoulder IR and external rotation (ER) and hip IR and ER. Participants were instructed to stretch in each direction to the point of maximum stretch where they felt a significant but painless stretch. Each position was held for 30 seconds and one set was completed for each direction [20]. Shoulder IR and ER ROM measurements were performed with the patients in the supine position (Figure 1, Figure 2). The starting limb position was 90° shoulder abduction, 10° horizontal shoulder adduction, and 90° elbow flexion on the measurement side [12]. One examiner performed the shoulder IR and ER passively on the measurement side, and the other measured the ROM using a goniometer with a bubble attached [12]. During shoulder rotation, the examiner's thumb palpated the participants' coracoid process from the initial movement [12]. The point at which the coracoid process was thrust into the examiner's thumb was palpated with the participants' shoulder passively rotated and was determined as the endpoint. Subsequently, the passive IR motion was stopped [12]. Palpation of the coracoid process thrust has been previously used to confirm compensation of the scapula and contribute to good measurement reliability [12,21,22]. The examiner was cautious to avoid manual compression of the humeral head and prevent abnormal movement of the glenohumeral joint [12]. The examiner verified the measurement with a bubble-level scale, wherein the basic axis of the goniometer was vertical and the axis of the movement arm was aligned with the long ulnar axis, and measured the angle between the basic and movement axes in one-degree increments.

Loss of glenohumeral IR (GIRLoss), glenohumeral ER insufficiency (GERInsufficiency), and total rotational motion deficit (TRMD) [23], commonly used in shoulder and elbow injury studies as indicators of dominant-shoulder rotation restriction, were calculated as follows: GIRLoss=(non-dominant-shoulder IR ROM)−(dominant-shoulder IR ROM), GERInsufficiency=(non-dominant-shoulder ER ROM)−(dominant-shoulder ER ROM), and TRMD=(non-dominant-shoulder total-rotation ROM)−(dominant-shoulder total-rotation ROM) [23]. The larger the positive value of these indices, the more restricted the dominant-shoulder rotation ROM [23].

**Figure 3:**
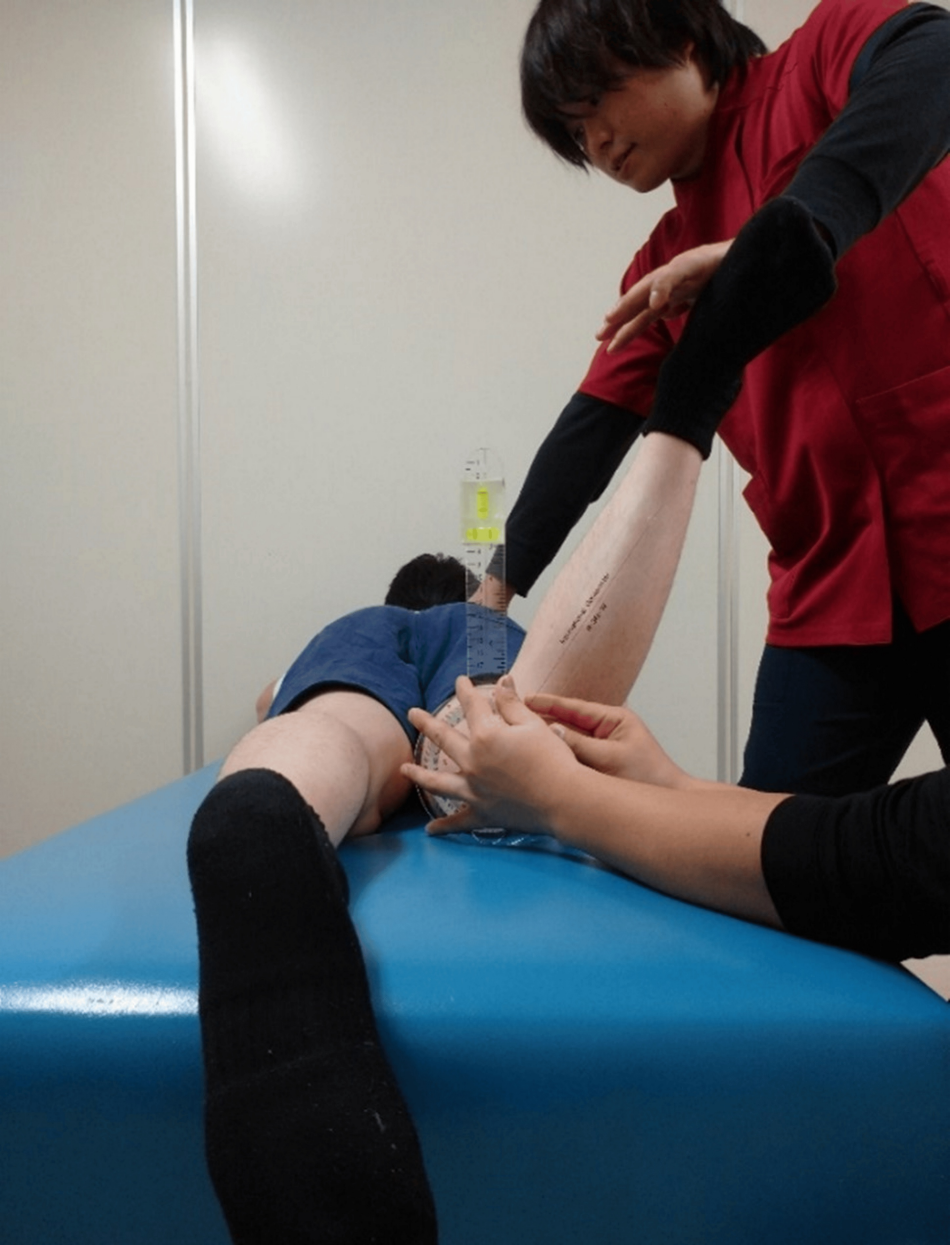
Range of motion measurement of hip internal rotation

**Figure 4:**
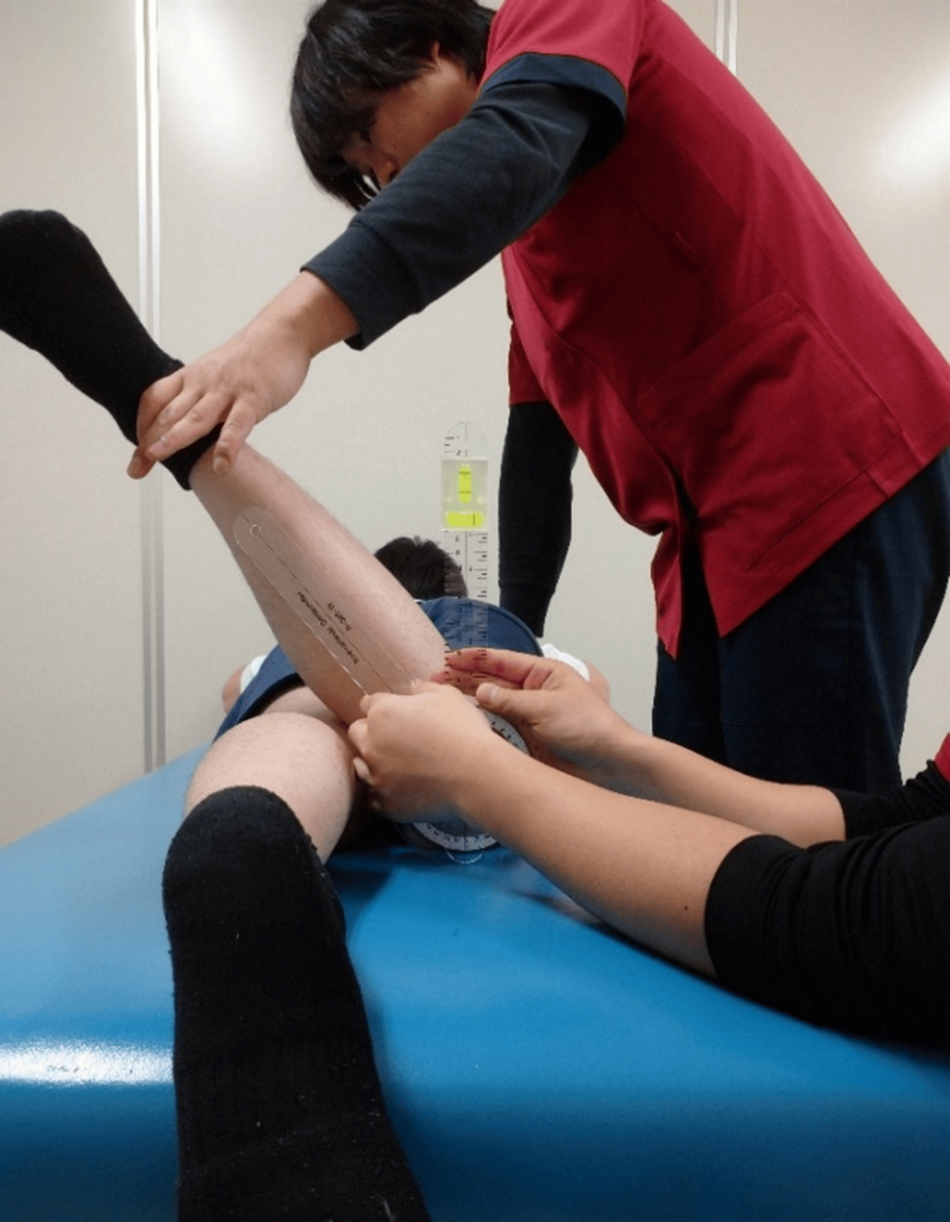
Range of motion measurement of hip external rotation

Hip IR and ER ROM were measured in the prone position (Figure 3, Figure 4). The starting limb position was the placement of the hip on the measurement side in intermediate IR/ER rotation and 90° knee flexion [3,21]. One examiner performed manual pelvic girdle immobilization and passively performed IR and ER of the hip being examined until reaching the endpoint [3,21]. Another examiner used a goniometer with a bubble attached to measure the ROM [3,21]. The examiner verified the measurements using a bubble-level scale, in which the basic axis of the goniometer was vertical and the axis of the movement side was aligned with the long axis of the lower leg. The endpoint ROM measurement was determined as either the point at which (1) the measurer perceived firm resistance during passive movement; (2) the measurer who was stabilizing the pelvic girdle confirmed the palpable onset of pelvic rotation; or (3) the participant experienced a strong but tolerable stretch, shortly before the occurrence of pain due to the rotatory movement [24,25].

Previous studies on the hip and LBP in athletes measuring hip rotation ROM have generally used the value as an index of hip rotation restriction [4,16,21,22]. Therefore, we used the measured hip rotation ROM as an index of rotation restriction for statistical analysis. Regarding both dominant- and non-dominant-hip rotation ROM, lower values indicated a more limited ROM.

Prior to this study, the intra-rater reliability of the IR and ER ROM of the shoulder and hip was verified using a test-retest method. This verification involved 10 male high school soft tennis players whose age, height, weight, and BMI were similar to those in the current study (these participants were not included in the current study). IR and ER ROM of the bilateral shoulder and hip were measured twice at a five-day interval using the same measurement methods. Intraclass correlation coefficient (ICC) (1,3) and the minimum detectable change with 95% confidence intervals (CIs; MDC95) were calculated [26]. MDC95 was calculated using the standard error of measurement (SEM) based on the following formula: SD/√2 of the difference between the two measurements; this was followed by the formula previously described (MDC95=SEM×1.96×√2) [26]. In this pilot study, the ICC (1,3) for shoulder IR and ER ROM was 0.98 (95% CI: 0.96-0.99) and 0.95 (95% CI: 0.89-0.98), respectively, and MDC95 was 2.4° and 2.6°, respectively. The ICC (1,3) for hip IR and ER ROM was 0.98 (95% CI: 0.96-0.99) and 0.97 (95% CI: 0.89-0.98), respectively, and MDC95 was 2.2° and 2.1°, respectively. These results indicated good intra-rater reliability for each ROM.

Statistical analysis

All statistical analyses were performed using the Excel statistical software package (Social Survey Research Information Co., Ltd., Tokyo, Japan). The participants were divided into LBP and non-LBP (NLBP) groups based on the presence of LBP lasting >2 weeks, which affected performance within the year before this research [3]. Basic characteristics, GIRLoss, GERInsufficiency, TRMD, and dominant- and non-dominant-hip rotation ROM angles were compared between the groups. GIRLoss, GERInsufficiency, and TRMD were calculated as the average of three repeated shoulder rotation ROM measurements using the abovementioned method. The average of three repeated measurements of hip rotation ROM between the groups for each dominant and non-dominant leg was obtained. Hip total-rotation (TR) ROM, calculated by summing the means of three repeated measurements of hip IR and ER ROM, was also compared between the groups for the dominant and non-dominant legs. Height, weight, GIRLoss, GERInsufficiency, TRMD, and dominant- and non-dominant-hip rotation (IR, ER, and TR) ROM were determined to be normally distributed using the Shapiro-Wilk test (P>0.05) and homogeneous using the Levene test (P>0.05). Therefore, comparative analyses were performed using Student's t-test. The number of years of competition experience, practice frequency per week, and total practice hours per week were determined to be non-normally distributed using the Shapiro-Wilk test. Therefore, comparative analyses were performed using the Mann-Whitney U test. Players' positions were compared using the χ² test. The significance level (P) for the univariate analyses was set at 5%.

Next, multiple logistic regression analyses were performed with the presence of LBP as a categorical variable (LBP, 1; NLBP, 0). Multiple logistic regression models were created as follows: Model 1 included dominant-shoulder IR restriction (GIRLoss), Model 2 included non-dominant-hip IR ROM, and Model 3 included both GIRLoss and non-dominant-hip IR ROM as the main explanatory variables to test the association between dominant-shoulder IR restriction and LBP, compare its suitability as an associated factor with non-dominant-hip IR restriction, and determine if either or both should be considered in an LBP association model based on model fit.

Height was fitted as a confounding factor in all models because it was identified as a factor associated with LBP in elite male high school soft tennis players [3]. Additionally, variables with P<0.1 in the between-group comparisons were fitted into all the models as confounders due to their potential influence on the objective variable [27]. In all models, Pearson's reserve correlation coefficients were calculated for the explanatory variables to be fitted, and variables with correlation coefficients of ≥0.7 were suspected to have multicollinearity [28]. The use of a variance inflation factor (VIF) of ≥5 as a criterion for suspecting multicollinearity has also been reported [25]. Therefore, we judged that multicollinearity was present when either of the two criteria was met. Considering variables determined to be multicollinear, only the variables with the highest priority were fitted based on the main purpose of this study. In each multiple logistic regression model, the regression coefficient, standardized partial regression coefficient, odds ratio (OR), and 95% CIs of the ORs were calculated, and a 5% significance level was set. The significance of each multiple logistic regression model was examined using the model χ^2^ test, and a model with P<0.05 was judged as a significant model. Model fit was examined using the Hosmer-Lemeshow test, and P≥0.05 was judged as a good model fit. The Akaike Information Criterion (AIC) and Bayesian Information Criterion (BIC) were used to compare the goodness-of-fit among multiple logistic regression models: the lower the AIC and BIC, the better the relative model fit [29].

The sample size was decided based on power analysis [25]. Using sample size calculation software (Power and Precision Version 4; BioSTAT Consultants, Inc., USA) for logistic regression analysis sample size design, we set the alpha error to 5% and the beta error to 10%. The detection power was confirmed as sufficient (>90%) for 167 or more cases as described above.

## Results

Overall, 59 and 117 participants were in the LBP and NLBP groups, respectively. The basic characteristics of participants showed no significant intergroup differences (Table 1). 

**Table 1:** Comparison of patient demographic characteristics between the LBP and NLBP groups Results are presented as mean (standard deviation) and frequency. Statistically significant difference: P<0.05 LBP: low-back pain; NLBP: non-low-back pain; BMI: body mass index

	All players (N=176)	LBP (n=59)	NLBP (n=117)	Effect size	P
Age, y	16.3 (0.7)	16.1 (0.7)	16.2 (0.6)	0.02	0.77
Height, cm	170.0 (6.4)	171.3 (6.4)	169.3 (6.3)	0.32	0.06
Weight, kg	58.4 (6.6)	59.2 (0.9)	58.0 (0.6)	0.04	0.60
BMI, kg/m²	20.2 (1.7)	20.2 (1.9)	20.2 (1.7)	0.03	0.40
Years of competition experience, y	5.9 (2.0)	5.9 (2.0)	5.9 (2.0)	0.01	0.92
Weekly practice frequency, d	6.3 (0.4)	6.3 (0.5)	6.2 (0.4)	0.03	0.67
Total weekly practice time, h	27.4 (1.9)	27.6 (2.0)	27.3 (1.8)	0.06	0.44
Positions (volleyer/baseliner), players	75/85	25/29	50/56	0.008	0.91

Table 2 shows the between-group comparison for GIRLoss, GERInsufficiency, TRMD, and dominant- and non-dominant-hip rotation ROMs. GIRLoss and TRMD were significantly higher in the LBP group than in the NLBP group (all P<0.01). The non-dominant-hip IR and TR ROMs were significantly lower in the LBP group than in the NLBP group (all P<0.01). Other measurements did not significantly differ between the groups.

**Table 2:** Comparison of shoulder and hip rotation range of motion Results are presented as mean (standard deviation). *Statistically significant difference: P<0.05 GIRLoss: loss of glenohumeral internal rotation; GERInsufficiency: glenohumeral external-rotation insufficiency; TRMD: total rotational motion deficit; LBP: low-back pain; NLBP: non-low-back pain

Direction of movement, °		LBP (n=59)	NLBP (n=117)	Effect size	P
GIRLoss		18.7 (11.7)	13.1 (12.6)	0.46	0.01*
GERInsufficiency		4.5 (8.4)	4.6 (8.8)	0.01	0.96
TRMD		14.2 (12.8)	8.3 (13.5)	0.45	0.01*
Hip internal rotation	Non-dominant leg	34.5 (11.3)	43.4 (8.6)	0.92	<0.01*
	Dominant leg	43.3 (9.7)	44.2 (8.8)	0.11	0.52
Hip external rotation	Non-dominant leg	48.9 (8.6)	49.2 (7.9)	0.04	0.81
	Dominant leg	49.8 (7.8)	49.0 (7.9)	0.09	0.23
Hip total rotation	Non-dominant leg	83.4 (12.5)	92.6 (10.3)	0.83	<0.01*
	Dominant leg	93.0 (11.5)	93.3 (8.8)	0.02	0.89

Table 3 summarizes the results of the multiple logistic regression analysis. In addition to height, TRMD and non-dominant-hip TR ROM, with P<0.1 in the univariate analysis, were used as confounding factors and candidate explanatory variables in all models. However, multicollinearity was judged between TRMD and GIRLoss, non-dominant-hip TR ROM, and non-dominant-hip IR ROM. Considering our study's main purpose, TRMD and non-dominant-hip TR ROM were excluded, and only height was included as a confounding factor in the explanatory variables of all the models. Model 1 (explanatory variables: GIRLoss+height), Model 2 (explanatory variables: non-dominant-hip IR ROM+height), and Model 3 (explanatory variables: GIRLoss+non-dominant-hip IR ROM+height) multiple logistic regression models were created. In Model 1, the ORs for GIRLoss and height were 1.04 (95% CI: 1.01-1.07; P<0.01) and 1.06 (95% CI: 1.00-1.12; P<0.01), respectively. In Model 2, the ORs for non-dominant-hip IR ROM and height were 0.90 (95% CI: 0.87-0.94; P<0.01) and 1.06 (95% CI: 1.00-1.12; P<0.01), respectively. In Model 3, the ORs for GIRLoss, non-dominant-hip IR ROM, and height were 1.06 (95% CI: 1.02-1.09; P<0.01), 0.90 (95% CI: 0.86-0.94; P<0.01), and 1.07 (95% CI: 1.01-1.14; P=0.02), respectively. In all models, the explanatory variables fitted were significantly associated with LBP, indicating that the models were significant and fitted well: the AICs (and BICs) for Models 1, 2, and 3 were 198.4 (207.6), 178.6 (187.9), and 168.8 (181.1), respectively, indicating that Model 3 was the best fit. As 59 cases were included in the LBP group, up to five explanatory variables were considered for inclusion in the logistic regression model. Therefore, the sample size of this study met the pre-designed sample size and included an appropriate number of explanatory variables.

**Table 3:** Multiple logistic regression analysis results *P<0.05, **P<0.01 CI: confidence interval; OR: odds ratio; RC: regression coefficient; SPRC: standardized partial regression coefficient; AIC: Akaike Information Criterion; BIC: Bayesian Information Criterion; GIRLoss: loss of glenohumeral internal rotation; ROM: range of motion; IR: internal rotation Model 1: y=(0.04×GIRLoss)+(0.05×height)−11.4 Model χ^2^ test: P=0.0023, Hosmer-Lemeshow test: P=0.18 Model 2: y=(0.098×non-dominant-hip IR ROM)+(0.06×height)−7.09 Model χ^2^ test: P<0.001, Hosmer-Lemeshow test: P=0.06 Model 3: y=(0.055×GIRLoss)+(−0.106×non-dominant-hip IR ROM)+(0.071×height)−9.63 Model χ^2^ test: P<0.001, Hosmer-Lemeshow test: P=0.22

	Explanatory variable	RC	SPRC	OR (95% CI)	P	AIC	BIC
Model 1	GIRLoss	0.04	0.52	1.04 (1.01-1.07)	<0.01**	198.4	207.6
	Height	0.05	0.38	1.06 (1.00-1.12)	0.033*		
Model 2	Non-dominant-hip IR ROM	-0.098	-1.02	0.90 (0.87-0.94)	<0.01**	178.6	187.9
	Height	0.06	0.39	1.06 (1.00-1.12)	0.037*		
Model 3	GIRLoss	0.055	0.69	1.06 (1.02-1.09)	<0.01**	168.8	181.1
	Non-dominant-hip IR ROM	-0.106	-1.12	0.90 (0.86-0.94)	<0.01**		
	Height	0.071	0.46	1.07 (1.01-1.14)	0.019**		

## Discussion

In this study, dominant-shoulder IR restriction (GIRLoss) was a major factor associated with LBP in elite male high school soft tennis players. Model 3, which included known associated factors such as non-dominant-hip IR ROM and height, also indicated that dominant-shoulder IR restriction was an independent factor associated with LBP. Specifically, the ORs suggest that every 1° increase in dominant-shoulder IR restriction was associated with a 1.06-fold increase in the risk of LBP. This supports the study's hypothesis that IR restriction in the dominant shoulder is associated with an increased risk of LBP.

Immediately before the impact of a serve or forehand stroke in lawn tennis, the angular velocity of the dominant-shoulder IR is the greatest [9]. Fleisig et al. examined the maximum angular velocity of the upper and lower limbs and trunks during the serving motion in male lawn tennis players and found that the maximum angular velocity of the dominant-shoulder IR was the highest, indicating a greater contribution to efficient impact [14]. Thus, the dominant-shoulder IR is critical in maximizing the energy from the lower limb to the ball impact in the second half of the serve and stroke motions. The dominant-shoulder IR motion also reduces excessive rotational torque on the lower lumbar spine during the stroke movement, reducing the mechanical load on the lumbar region [16]. Serve and stroke movement characteristics in soft tennis are similar to those in lawn tennis [3], and their mechanical complements are similar. In soft tennis players, dominant-shoulder IR restriction may contribute to LBP due to the loss of the original mechanical contribution of the dominant-shoulder IR motion, resulting in excessive rotational loading at the lower back. The only study that reported an association between shoulder ROM and LBP was by Narita et al., who assessed shoulder elevation ROM during swimming dives [30]. They reported that shoulder elevation ROM restriction may increase the compensatory load due to excessive lumbar extension in the lower back and contribute to LBP [30]. However, no reports exist regarding shoulder rotation restriction and LBP in other sports. 

A previous report showed that non-dominant-hip IR restriction was a significant factor associated with LBP in elite male high school soft tennis players [3], and Model 2 in the present study showed similar results. In tennis competitions, non-dominant-hip IR motion during the stroke and serve movements plays a fundamental and primary role in efficiently transferring energy from the lower limb to the upper limb [3]. Furthermore, in elite male high school soft tennis players with non-dominant-hip IR restriction, compensatory loading in the lower back may occur and contribute to LBP [3]. Similar reports on other sports in which the rotational component of the body is important have suggested the need for improvement in non-dominant-hip IR restriction as a preventive measure for LBP [7,8,10,19]. Notably, in Model 3, which included dominant-shoulder IR restriction in addition to non-dominant-hip IR restriction, a known major associated factor of LBP, dominant-shoulder IR restriction was identified as an independent associated factor. The rationale that both non-dominant-hip and dominant-shoulder IR restrictions independently contribute to LBP is significant. This study highlights the importance of addressing dominant-shoulder IR restriction to prevent LBP in elite male high school soft tennis players.

Model 1, with dominant-shoulder IR restriction as the main explanatory variable, had a poorer fit than Model 2, which used non-dominant-hip IR restriction as the main explanatory variable, indicating that non-dominant-hip IR restriction may be more important than dominant-shoulder IR restriction in developing a model of factors associated with LBP. Generally, the ascending kinetic chain from the lower limbs to the upper body is extremely important for efficient hitting and throwing movements [7,8,10,19]. In tennis competitions, non-dominant-hip IR motion is also an important kinetic chain starting point for the efficient transfer of energy to the upper limb for effective strokes and serves [3,17]. Although the dominant-shoulder IR is also mechanically important for efficient ball impact [14], it occurs later in the kinetic chain process and may not be as important as the non-dominant-hip IR in terms of its contribution to the kinetic chain. Therefore, non-dominant-hip IR restriction may more likely increase the relative compensatory load on the lower back and contribute to LBP. However, Model 3, with both dominant-shoulder and non-dominant-hip IR restrictions as explanatory variables, showed the best fit, suggesting the inclusion of these variables as factors associated with LBP. Understanding the mechanism of LBP development is important, considering that the higher the degree of both restrictions, the higher the compensatory load on the lower back and the higher the symptomatic risk of LBP.

Therefore, improving dominant-shoulder IR restriction to prevent LBP in elite male high school soft tennis players is important. Furthermore, concurrent improvement in non-dominant-hip IR restriction may be a more effective measure for preventing LBP.

This study has some limitations. First, it was cross-sectional, making it difficult to establish a causal relationship between dominant-shoulder IR restriction and LBP. Players may have developed LBP before high school and may not accurately recall its onset. To clarify causality, a randomized controlled trial testing whether interventions for shoulder IR restriction can prevent LBP is necessary. Second, LBP presence was assessed using a self-administered questionnaire, potentially introducing recall bias. Third, the study did not use a physician's diagnosis, orthopedic tests, or imaging to confirm LBP. Future studies should include these criteria. Fourth, the mechanical relationship between dominant-shoulder IR restriction and the lower back should be verified using a three-dimensional motion analyzer.

## Conclusions

Dominant-shoulder IR restriction was associated with LBP in elite male high school soft tennis players. Additionally, including non-dominant-hip and dominant-shoulder IR restrictions as factors may enhance the development of an optimal multiple logistic regression model for LBP and guide effective prevention measures. The clinical significance of this study lies in highlighting the need for LBP prevention measures in these athletes, particularly concerning dominant-shoulder IR restriction. The findings also provide a rationale for emphasizing both dominant-shoulder and non-dominant-hip IR restrictions in LBP prevention. 
